# Handwriting and Motor-Related Daily Performance among Adolescents with Dysgraphia and Their Impact on Physical Health-Related Quality of Life

**DOI:** 10.3390/children9101437

**Published:** 2022-09-21

**Authors:** Liat Hen-Herbst, Sara Rosenblum

**Affiliations:** 1 Department of Occupational Therapy, School of Health Sciences, University of Ariel, Ariel 4077625, Israel; 2 Department of Occupational Therapy, Faculty of Welfare and Health Sciences, University of Haifa, Mount Carmel, Haifa 3498838, Israel

**Keywords:** handwriting, dysgraphia, motor-related daily functions, adolescent, quality of life

## Abstract

Knowledge is limited about dysgraphia in adolescence and its association with daily motor-related daily performance and health-related quality of life (HRQOL). This study aimed to (1) compare and (2) examine correlations between handwriting measures, motor-related daily performance, and HRQOL of adolescents with and without dysgraphia and (3) examine the contribution of motor-related daily performance and handwriting measures to predict their physical HRQOL. There were eighty adolescents (13–18 yr): half with dysgraphia and half matched controls without dysgraphia per the Handwriting Proficiency Screening Questionnaire and Handwriting Legibility Scale participated. They copied a paragraph script onto a paper attached to the Computerized Penmanship Evaluation Tool digitizer and completed the World Health Organization Quality of Life Questionnaire-brief version and the Adult Developmental Coordination Disorder Checklist (ADC). We found significant between-group differences in motor-related daily performance, handwriting measures, and HRQOL and significant correlations between HRQOL and handwriting process measures and motor-coordination ability. Handwriting measures predicted 25%, and the ADC A and C subscales 45.6%, of the research group’s physical QOL domain score variability. Notably, the control group’s current perceptions of their motor-coordination performance (ADC-C) predicted 36.5% of the variance in physical QOL. Dysgraphia’s negative effects during childhood and adolescence may reduce adolescents’ HRQOL now and into adulthood.

## 1. Introduction

Despite the expanding use of technology, handwriting remains an everyday school activity [1]. As a fundamental literacy component, the ability to write is vital to succeeding in school and the majority of workplace environments [2]. The National Handwriting Association [3] proposed that the act of handwriting is a visually symbolic format for individuals to represent language and concepts physically and permanently. The handwriting task encompasses sharing learning and ideas. This single activity involves perceptual, cognitive, language, and motor skills. As an art form, the written text allows for self-expression. Typically, handwriting is only taught in the early grades [4]; thus, children most often acquire handwriting skills in their first 3 years of school [5]. 

This study focuses on adolescents’ handwriting. The word *adolescence* derives from the Latin *adolescere*—to grow up [6]. This phase of life, which stretches between childhood and adulthood, encompasses elements of biological growth and major social role transitions. The timing of these development patterns varies across time and place [7]. According to Sawyer et al. [7], age definitions are always arbitrary. In this study, we use an expanded, more inclusive definition of adolescence as 10 to 24 years of age to align more closely with contemporary patterns of adolescent growth and popular understandings of this life phase.

Handwriting is an essential gateway to academic success for high-school students because it supports classroom participation [8]. According to Miller et al.’s [9] systematic literature review, difficulties in this area could affect the students’ self-esteem, school behavior, and grades. Furthermore, previous empirical studies associated specific learning disorders (including handwriting difficulties) and social–emotional and behavioral problems with significant effects on well-being (e.g., [10]). Difficulties in written expression, spelling, and handwriting are often considered manifestations of dysgraphia. The Diagnostic and Statistical Manual of Mental Disorders (5th ed.; *DSM–5*) termed dysgraphia a “specific learning disorder with impairment in written expression” [11]. According to Döhla et al. [12], between 7% and 15% of children of school age exhibit some developmental writing deficit, including poor legibility (e.g., [13]). In addition, students with dysgraphia may write slowly (e.g., [14]) and experience pain and fatigue while writing [15]. Dysgraphia disrupts writing skills acquisition and significantly frustrates students emotionally. Research has associated it negatively with academic functioning. It disrupts students’ abilities to record and communicate ideas, complete schoolwork, learn, and demonstrate what they learned [16,17]. For many students, these difficulties persist over time. Handwriting difficulties that originate in childhood may continue in young adulthood (e.g., [18]). Thus, dysgraphia’s detrimental effects are not limited to children. Adolescents and adults with significant writing deficits struggle with daily tasks requiring writing skills and face limited potential for career advancement or choices [2,19].

Various models describe the complexity of handwriting, and some outline the motor processes involved in the skill (e.g., [20]). Previous studies showed that individuals with motor-function difficulties, as in developmental coordination disorder (DCD), often show handwriting difficulties [21,22,23] and suggested that the process of handwriting requires multiple motor functions. Studies correlated difficulties in anticipatory planning, motor control, and movement-skills automatization difficulties with students’ handwriting difficulties. The difficulties manifested in both the handwriting product, specifically letter formation and organization of the text spatially on the paper (i.e., legibility) [24,25], and the handwriting process, which produced slower writing and more pauses than typically developed students [25,26]. In addition, fine-motor sequential movement and finger dexterity have been linked with elementary school students’ (e.g., [27]) and adults’ [28] handwriting performance. However, according to Tal-Saban and Weintraub [29], actual knowledge of the characteristics of students with dysgraphia or their associated motor functions is lacking.

Handwriting is one of many other daily functions that requires motor learning and execution. Hence, one can expect that the motor difficulties related to handwriting performance also may affect other motor-based daily functions [29]. Along with handwriting problems, common motor-skills difficulties and motor-related daily function difficulties experienced in early childhood endure into young adulthood and might restrict almost every life activity [29,30]. The persistent, complex impacts of the childhood difficulties that continue into adolescence—and new difficulties acquired in adolescence—accentuate the need to assess adolescents’ motor-coordination difficulties as reflected in their daily function. However, most research on DCD examined populations of affected children rather than adolescents and adults. In addition, few coordination assessments specifically for the adolescent population effectively capture a wide range of motor-coordination issues, and a gold standard is absent. 

Furthermore, each existing assessment has limitations based on age, abilities covered, the time required, or focal areas [31]. According to Kempert et al. [32], the available assessments for adolescents with suspected DCD are not sensitive enough to detect their impairments.

The most highly cited questionnaire [33], the Adult Developmental Coordination Disorder/Dyspraxia Checklist (ADC) [34], was designed to consider the range of motor, organization, attention, and social difficulties commonly associated with DCD. The ADC provides information about individuals’ motor coordination in daily situations and as experienced as children. It also addresses adults’ motor-skill-related self-esteem as they and others perceive them. The ADC’s psychometric properties show good internal consistency and construct, concurrent, and discriminant validity among adults [34,35,36]. Kirby et al. [35] correlated motor-coordination difficulties with adults’ reduced general, physical, and mental health. 

Health-related quality of life (HRQOL) is the quality of life relative to one’s health or disease status [37]. The HRQOL is a multidimensional construct. It encompasses emotional well-being, interpersonal relations, physical well-being (health, activities of daily living, and leisure), material well-being (e.g., financial status, employment, and housing), personal development (i.e., education, personal competence, and performance), social inclusion, self-determination, and legal and human rights [38]. Leading health organizations have identified HRQOL as a goal for all people across all life stages [39,40].

Our study aims to elaborate the knowledge about dysgraphia in adolescence by exploring the relationships between handwriting performance and motor-coordination abilities as reflected in children’s and adolescents’ daily functioning and how they predict HRQOL. This study specifically aims to (1) compare the motor-coordination performance, handwriting process, and HRQOL of adolescents with and without dysgraphia, (2) examine correlations between the motor-coordination performance, handwriting process, and HRQOL of adolescents with and without dysgraphia, and (3) review the motor performance and handwriting measures’ contributions to predicting physical HRQOL among adolescents with and without dysgraphia.

## 2. Materials and Methods

### 2.1. Participants

Eighty adolescent (13–18 yr) native Hebrew speakers and writers who were students in the regular public educational system participated in this study. Their socioeconomic levels ranged from low to high, according to the Central Bureau of Statistics in Israel criteria [41]. Of the participants, we defined half (40) as having dysgraphia based on their Handwriting Proficiency Screening Questionnaire (HPSQ-C/HPSQ) self-reports [42,43]. An expert occupational therapist approved their report by the Handwriting Legibility Scale (HLS) assessment of their written product’s legibility [44]. The other half (40) were gender- and age-matched controls without dysgraphia. This control group included classmates of the adolescents referred by the research group participants. They were defined as controls based on their self-reported HPSQ-C/HPSQ scores. Exclusion criteria were known autistic, neurotic, or emotional disorders; neurological diseases; or physical disabilities, all according to parental reports. The participants’ sociodemographic information is summarized in Table 1. We found no significant between-group differences in gender, age, mothers’ years of education, socioeconomic status, or dominant hand. 

### 2.2. Instruments for Participant Selection

#### 2.2.1. Handwriting Proficiency Screening Questionnaire

The 10-item self-report HPSQ-C [42] is a reliable, valid questionnaire designed to identify children with handwriting difficulties. The HPSQ-C uses the same questions and scoring as the HPSQ [43] but is a self-report. The 10 items are grouped into three factors: legibility, physical and emotional well-being, and performance, and the final score comprises the sum of all items. Higher sums indicate poorer handwriting performance. The HPSQ-C cutoff score for handwriting deficiency is the same as the HPSQ [43]: 14 or more.

#### 2.2.2. Handwriting Legibility Scale

The HLS [44] provides a fast, easy-to-use, and holistic handwriting–legibility assessment. It focuses on the text layout, letter formation, alterations, and an overall impression of the legibility as the effort required to read the handwriting. Instructions for scoring emphasize gaining an “overall impression” of each criterion when deciding how to score for each component. A 5-point Likert scale (1–5, good–poor) is used to assess each criterion, providing total scores from 5 to 25. Higher scores reflect poorer legibility.

### 2.3. Instruments for Comparing between Groups

#### 2.3.1. Computerized Penmanship Evaluation Tool (ComPET: Previously, POET) [13]

Participants performed a writing assignment: They used a wireless electronic pen with a pressure-sensitive tip (Model GP-110) to copy a paragraph onto a paper attached to a Wacom Intuos II x-y digitizing tablet (404 × 306 × 10 mm). These are part of the ComPET system that records the exact performance time (s), mean pressure on the writing surface (nonscaled units 0–1024), mean stroke height (mm), and mean stroke width (mm). After considering previous results [45,46], we concentrated on these temporal and spatial measures of the written stroke: (1) mean stroke duration (MSD, i.e., performance time in s), (2) mean stroke width (MSW, i.e., the whole stroke width on the x-axis, in mm), and (3) mean stroke height (MSH, i.e., the whole stroke height, in mm).

#### 2.3.2. Adult Developmental Coordination Disorders/Dyspraxia Checklist

The self-reported ADC questionnaire [34] assesses the individual’s motor function in an everyday context. It includes items related to motor organization in space and time while performing daily living activities, self-care, everyday vocational or academic activities (e.g., driving or writing), hobbies, and social participation. Evidence from these items may support the existence of DCD according to the *DSM-IV* criteria and its probable existence based on current *DSM-5* [11] criteria. The ADC usually takes 15 to 20 min to complete. It is composed of three subscales: (A) the individual’s difficulties experienced as a child (versus problems acquired in adulthood), (B) the individual’s perception of their performance, and (C) others’ feelings about the individual’s current performance. Respondents rank the frequency of each difficulty’s occurrence on a Likert scale of 1 (*never*), 2 (*sometimes*), 3 (*frequently*), or 4 (*always*). Each scale’s scores are summarized. Lower scores indicate better performance. The ADC is feasible for distinguishing adults with coordination disorders from those without [36]. Kirby et al. [34] reported an internal reliability of 0.95 overall with the three subscales ranging from 0.87 to 0.91 among adults. In our study, the questionnaire Cronbach’s alpha was 0.94.

#### 2.3.3. World Health Organization Quality of Life Questionnaire, Brief Version 

The WHOQOL-BREF [45] consists of 26 items divided into four domains, each rated on a 5-point Likert scale: physical health, psychological, social relationships, and environment. The physical domain addresses activities of daily living, dependence on medicinal substances and medical aids, energy and mobility, pain and discomfort, sleep and rest, and work capacity. Facets of the psychological domain are negative and positive feelings; appearance and body image; religion, spirituality, and personal beliefs; self-esteem; and memory, concentration, thinking, and learning. The QoL social domain relates to satisfaction with personal relationships and support structure. Finally, the QoL environmental domain addresses physical safety, security, home environment, and health and social care. In each domain, higher scores represent better HRQOL. The WHOQOF-BREF has been used in previous research among adolescents (e.g., [47,48,49]) with Cronbach’s alpha values above 0.85. In our study, the questionnaire’s Cronbach’s alpha was 0.72 for the physical scale and 0.85 for the total score.

### 2.4. Procedure

We published advertisements on social media inviting adolescents with handwriting difficulties to participate in the study. Interested adolescents contacted the research coordinators by phone; they and their parents received detailed information about the study. Both the parents and children signed online consent forms. The parents completed the demographic questionnaire, providing sociodemographic data and information about the adolescents’ health status, which we evaluated according to the inclusion criteria. Parents of volunteers who met the inclusion criteria then completed an online questionnaire addressing the participants’ functional and developmental backgrounds; the adolescents completed an online version of the HPSQ-C. Additionally, each sent a sample (at least 20 complete lines) of free-writing script. These samples were assessed by a professional occupational therapist using the HLS to validate the score of the self-reported questionnaire (HPSQ-C). We divided participants into two groups. Half (40) had dysgraphia based on both their self-reported HPSQ-C (≥14) and high HLS scores. The other 40, defined as without dysgraphia, served as age- and gender-matched controls. The data collector met each participant in the participant’s home. During this meeting, the adolescents copied a paragraph on the digitizer and completed the WHOQOL-BREF and ADC.

### 2.5. Data Analysis

We conducted descriptive statistics for all measures, presenting means and standard deviations for continuous data and frequencies and percentages for categorical data. We used paired *t* tests for intragroup comparisons and chi-square tests for discrete variables. Normality tests were applied, and normal distribution was found for all measures (HPSQ-C, HLS, ComPET, ADC, and WHOQOL-BREF). *t* tests were conducted to assess differences in total scores. For calculations of effect sizes, Cohen’s d and η^2^ were conducted. Cohen’s d was calculated using the two groups’ means and standard deviations in the formula: Cohen’s d = (M_2_ − M_1_)/SD_pooled;_ where SD_pooled_ = √((SD_1_^2^ + SD_2_^2^)/2), 0.10 was considered small effect, 0.30 considered medium effect, and 0.50 considered large effect [50]. The formula to calculate eta squared was η^2^ = SS_effect_/SS_total_. In interpreting the eta-squared values, 0.01 was considered small effect, 0.06 considered medium effect, and 0.14 or higher considered large effect size [50]. Multivariate analysis of variance (MANOVA) was used to test the ADC and WHOQOL-BREF subscales, and Pearson tests to examine correlations between the outcome measures. Following the correlation results, we used linear regression model identifying relationships between two variables by fitting a linear equation to observed data. We used this method to test the contribution of each handwriting-process measure and each ADC subscale toward predicting physical HRQOL. A significance level of 0.05 was set for all statistical tests.

## 3. Results

### 3.1. Screening (HPSQ-C and HLS) 

As shown in Table 2, participants with handwriting difficulties had significantly higher scores (representing poorer handwriting performance) than the controls in the total HPSQ-C and HLS scores.

### 3.2. Comparison between Groups, Handwriting Performance (ComPET)

We implemented a MANOVA to compare the groups across the handwriting performance’s MSD, MSH, and MSW. The results showed significant group differences, *F*(2,77) = 15.8, *p* < 0.001, η^2^ = 0.29. Table 3 presents the post hoc results revealing significant group differences in all measures and a high eta for stroke performance time. These results indicated significantly higher, wider strokes (premature handwriting) and slower performance (higher mean-stroke duration) among research group participants.

### 3.3. Comparison between Groups, Motor-Coordination Performance (ADC)

As shown in Table 4, the significantly higher scores of participants with dysgraphia represented poorer motor coordination than the controls in all three ADC scales and total scores.

### 3.4. Comparison between Groups, QOL (WHOQOL-BREF)

Table 5 shows that participants with dysgraphia had significantly lower scores (representing lower self-perception of QOL) than the controls in the psychological and environmental scales and total QOL scores.

### 3.5. Correlations between Motor-Related Daily Performance, Handwriting Measures, and QOL

For all participants, we found significant correlations between the ADC total score and MSD; r = 0.294, *p* < 0.01, MSH; r = 0.374, *p* < 0.01, and MSW; r = 0.254, *p* < 0.05, meaning that motor-related daily performance was associated with the handwriting process measures. 

Correlations between all handwriting measures, ADC subscales, and QOL measures were analyzed. Table 6 presents only the significant correlations found for each group separately. In the group of adolescents with dysgraphia, we found significant correlations between two handwriting measures (MSD and MSH) and physical QOL. The ADC-C subscale (others’ current feelings about the individual’s performance) significantly correlated with all HRQOL scales except the social scale. As also found among the adolescents with dysgraphia, the ADC-C subscale significantly correlated with the physical QOL and HRQOL total scores (Table 6).

### 3.6. Predicting Physical QOL by Handwriting Measure

According to the finding that handwriting measures were correlated only with physical QOL, we then tested the handwriting-process measure and ADC subscale contributions toward predicting physical HRQOL. The results for adolescents with dysgraphia produced a model that included MSH and MSD as significant predictors of the QOL physical domain by handwriting measures. Mean stroke height and duration contributed 25% of the variance, *F*(1,38 ) =  6.14, *p*  =  0.005. In the control group, the handwriting measures were significant predictors of the physical QOL domain (Table 7).

### 3.7. Predicting Physical QOL by ADC Subscale

Predicting the QOL physical domain by the ADC subscales for adolescents with dysgraphia yielded two models. The first included ADC-C (others’ feelings about the individual’s performance) as a significant predictor. It accounted for 26.6% of the variance, *F*(1,38) = 15.161, *p* < 0.001. A second model combined the ADC-A (performance as a child) as a significant predictor contributing an additional 21.8% of the variance, *F*(1,38) = 14.217, *p* < 0.01. Predictions among the control group yielded only one model, in which the ADC-C accounted for 38% of the variance, *F*(1,38) = 23.41, *p* < 0.001 (Table 8).

### 3.8. Predicting Physical QOL by Handwriting Measure and ADC Subscale among Adolescents with Dysgraphia

Prediction of the physical HRQOL for adolescents with dysgraphia yielded a model that included MSH as a significant predictor accounting for 13% of the variance, F(1,38) = 5.65, *p* < 0.05; MSD as a significant predictor of an additional 12% of the variance, F(1,37) = 5.89, *p* < 0.05, and ADC-C (current feelings about the individual’s performance as reflected by others) as a significant predictor of an further 13% of the variance, F(1,36)  = 7.57, *p*  < 0.01 (Table 9).

## 4. Discussion

This study intended to expand the limited knowledge regarding poor motor coordination and difficulties in handwriting performance as expressed in daily activities among adolescents and to examine relationships between the adolescents’ HRQOL and these measures. Although previous studies had described the impact of dysgraphia on daily functions and well-being among children [51] and adults [29], the knowledge about adolescents with dysgraphia is limited. Therefore, this study’s contribution is significant and vital.

The results of this study indicate that adolescents with dysgraphia show significantly inferior handwriting product legibility and performance time. These results support prior studies on younger children’s (e.g., [46]) and university students’ (e.g., [36]) handwriting processes.

According to our results, adolescents with dysgraphia perceived their functional motor-coordination abilities during childhood and adolescence as poorer than the controls. These results align with previous results among school-aged children (e.g., [52]), higher education students [29], and adults [30]. Similar to those studies, our results promote the understanding that handwriting difficulties and other motor-coordination restrictions experienced as children are not limited to childhood. They continue into adolescence and adulthood and persistently affect daily activities.

This study also found that adolescents with dysgraphia felt lower HRQOL across multiple domains. Although this finding was not significant in physical and social domains, it was significant for the psychological and environmental domains. Given the motor-based nature of the handwriting process, these results at first glance might seem surprising. They could suggest that, despite manifesting physically, dysgraphia’s adverse effects on adolescents are more pronounced in the emotional domain and interactions with the environment. A plethora of prior studies explored the effects of motor-coordination difficulties on psychological domains. For instance, they looked at behavior, emotional health, cognitive function, self-concept, and self-efficacy (e.g., [51,53,54]). In interviews with children with DCD, Zwicker and colleagues [55] asked about handwriting difficulties. Their participants had attempted to master the fine-motor skills required to succeed scholastically, but those attempts often came at emotional costs. Those researchers concluded that these findings indicated an overlap in the physical and psychological QOL domains that can be impacted in children with DCD and handwriting difficulties [55]. 

Regarding the interaction with the environment, previous studies found that assisting children with educational curriculum may decrease the stress associated with writing [56,57]. Further, the environment’s adaptive nature crucially enables individuals to enjoy good health and participate [58]. Hence, significant differences between groups in the environmental domain could be expected. Finally, our result of no significant differences in the social domain supports previous results that reported that children with specific learning disorders viewed their familiar and interpersonal social competence in a generally positive light [59]. Given the contrasting published evidence on difficulties in relationships with peers among children with specific learning disabilities [60] and lower HRQOL in the social domain among adults with suspected DCD [30], our results need to be further investigated in future studies with larger samples of adolescents with dysgraphia.

As expected, significant correlations were found between the handwriting performance measures and the motor-based daily performance measures. These results support previous studies showing that individuals with motor-function difficulties, as in DCD, often show handwriting difficulties [21,22,23]. As expected, correlations between the handwriting performance measures and HRQOL were found only for the group with dysgraphia. Reasonably, handwriting performance would not correlate with or affect the HRQOL of individuals without handwriting difficulties.

Significant correlations were also found between the ADC-C (others’ feelings about the individual’s motor-related daily performance) and the HRQOL physical domain in both groups. These results could be expected based on the physical/motor manifestations of DCD. More so, the ability to perform various motor actions, including the coordination of fine- and gross-motor skills, is necessary to master daily life activities [61]. The HRQOL reflects the degree to which a person can participate with or without assistance socially, physically, and emotionally [58]. The fact that correlations were found for participants from both groups with the ADC-C, which assesses the self-perceived feelings of others, can be explained by the heightened sensitivity that characterized adolescents with and without difficulties to social interactions with others [7]. More than children or adults, adolescents have been found to be more sensitive to peer acceptance, rejection, and approval [62,63]. Furthermore, high-quality social interactions with peers appear to protect against mental health problems and strengthen adolescent resilience and well-being [64]. Thus, the correlations found in our study between these two measures were expected. 

This study’s prediction analysis of the physical HRQOL yielded different models for each group. The handwriting measures predicted 25% of the physical HRQOL variance among the adolescents with dysgraphia but were not significant for the control group. Furthermore, predicting the physical domain by the ADC subscales revealed two models among adolescents with dysgraphia. The ADC-C predicted 26.6% and, together with the ADC-A (as a child), 45.6% of the variance in physical HRQOL. Among adolescents without handwriting difficulties, only the ADC-C was found to be a significant predictor for physical HRQOL: It explained 36.5% of the variance in this domain. Altogether, these results emphasize the tremendous impact of self-perceived competence in daily activities on emotional feelings and self-perceived QOL among adolescents with and without difficulties. 

We noted two more factors as significant predictors of the physical HRQOL only among the group with dysgraphia: handwriting process measures and functional motor-coordination difficulties the individual experienced as a child (ADC-A). These results highlighted, to the best of our knowledge, for the first time among adolescents, the emotional toll of unsuccessful attempts to perform complex daily motor-coordination activities. They emphasize the long-term impact of self-perceived difficulties in handwriting and other daily activities, demonstrating that the difficulties experienced as a child affect an individual’s HRQOL during adolescence. 

Finally, a linear regression was conducted with the handwriting measures and the ADC-C to predict physical HRQOL among the group of adolescents with dysgraphia. The results showed that process measures of handwriting, together with poor motor performance, explained 38% of the variance in this domain. These results reinforce previous studies’ findings regarding the importance of adolescents’ successful participation. Participation, the extent and nature of a person’s involvement in meaningful life situations at home, work, school, or community, is a major factor in the WHO’s International Classification of Functioning Disability and Health for Children and Youth [40]. Participation includes engaging in social, arts, or sporting activities; achieving academically; and integrating into the community. It encompasses milestones characteristic of emerging adulthood (e.g., higher education, careers, and independent living). Children and adolescents develop their competencies while participating; they learn essential life skills and form relationships with others [65]. As with our findings, previous studies linked participation to QOL and life satisfaction (e.g., [66]). 

Further research is needed to understand the repercussions of handwriting difficulties on adolescents’ academic and nonacademic outcomes. However, the significant relationships between QOL and handwriting performance in this study underscore the value of developing tools and services that advance the well-being of adolescents with dysgraphia through policies, programs, and interventions.

There are limitations to consider in this study. First, this study included a relatively small sample and should be replicated with a larger sample size. Second, differing motor deficits and handwriting performance may relate to co-occurring disorders, including reading and ADHD, that the participating students with dysgraphia may have. Several participants also may have had DCD but did not self-report it as a childhood diagnosis. Therefore, future studies of handwriting performance should examine these groups separately. Third, the limited availability of standardized assessment tools to measure handwriting legibility and coordination, specifically among adolescents, forces researchers to choose a tool, despite the limitations of age, abilities covered, the time required, or focal areas, as in this study. Future research should aim at suggesting more standardized tools for adolescents.

## 5. Conclusions

Dysgraphia may persist into adolescence and reduce HRQOL. Difficulties in handwriting correlated with difficulties performing other daily motor-coordination-based activities as a child and currently as an adolescent. The results highlight the emotional aspects of the difficulties in handwriting and other daily activities. Hence, we recommend multidisciplinary interventions that include occupational therapists, psychologists, and relevant health providers. Emotional support and promoting capabilities, performance, and participation in meaningful life situations and environments may minimize the adverse effects of dysgraphia and enhance the adolescents’ participation and quality of life.

## Figures and Tables

**Table 1:** Participants’ sociodemographic characteristics.

Characteristics		Dysgraphia (*n* = 40) M (SD)	Controls (*n* = 40) M (SD)	*t* (78)
Age		15.6 (1.33)	15.6 (1.49)	0.08
Mothers’ YOE ^a^		16.5 (2.74)	17.1 (3.39)	0.87
		Frequency (%)	Frequency (%)	η^2^
Gender	Boys	34 (85)	29 (72.5)	1.87
	Girls	6 (15)	11 (27.5)	
SEL ^b^	Low	1 (2.5)	0	4.03
	Average High	11 (27.5) 28 (70)	5 (12.5) 35 (87.5)	
Dominant hand	Right	31 (77.5)	33 (82.5)	0.31
	Left	9 (22.5)	7 (17.5)	

^a^ Years of education; ^b^ socioeconomic level.

**Table 2:** Comparison between groups for screening measures.

Measure	Dysgraphia (n = 40) M (SD)	Controls (n = 40) M (SD)	*t* (78)	d
HPSQ-C Total score	20.00 (4.46)	8.13 (3.63)	13.07 ***	2.95
HLS Total score	13.59 (4.16)	7.15 (2.19)	8.64 ***	1.93

*** p < 0.001.

**Table 3:** Comparison between groups for handwriting performance measures.

Measure ^a^	Dysgraphia (n = 40) M (SD)	Controls (n = 40) M (SD)	*t* (78)	η^2^
MSD	0.31 (0.09)	0.23 (0.04)	22.46 ***	0.23
MSH	0.31 (0.14)	0.25 (0.06)	5.11 *	0.07
MSW	0.19 (0.07)	0.17 (0.04)	4.03 *	0.05

^a^ MSD, mean stroke duration (s); MSH, mean stroke height (mm); MSW, mean stroke width (mm).* *p* < 0.05; *** *p* < 0.001.

**Table 4:** Comparison between groups in motor-coordination performance measures.

Measure ^a^	Dysgraphia (*n* = 40) M (SD)	Controls (*n* = 40) M (SD)	*F*(1,78)	η^2^
ADC-A	2.04 (0.48)	1.39 (0.34)	48.38 ***	0.38
ADC-B	1.94 (0.40)	1.36 (0.34)	47.55 ***	0.38
ADC-C	1.94 (0.32)	1.56 (0.28)	30.98 ***	0.28
ADC-Total	1.96 (0.32)	1.47 (0.24)	*t*(78) = 7.84 ***; *d* = 1.73	

^a^ ADC-A, childhood difficulties; ADC-B, self-perceived performance, ADC-C, others’ perceptions of current performance. *** *p* < 0.001.

**Table 5:** Comparison between groups in QOL measures.

Measure	Dysgraphia (n = 40) M (SD)	Controls (n = 40) M (SD)	*F*(1,78)	η^2^
Physical	3.98 (0.66)	4.14 (0.50)	1.58	0.02
Psychological	3.69 (0.53)	4.00 (0.42)	7.89 **	0.09
Social	4.14 (0.72)	4.04 (0.70)	0.39	0.01
Environmental	3.92 (0.58)	4.16 (0.33)	5.77 *	0.07
Total	3.93 (0.47)	4.14 (0.31)	*t* (78) = 2.35 * *d* = 0.53	

* *p* < 0.05; ** *p* < 0.01.

**Table 6:** Correlations between motor coordination, handwriting measures, and health-related quality of life (HRQOL).

	Dysgraphia			Control		
Measure	MSD	MSH	ADC-C	MSD	MSH	ADC-C
Physical QOL	−0.38 **	−0.36 *	−0.53 ***			−0.61 ***
Psychological QOL			−0.45 **			
Social QOL						
Environmental QOL			−0.36 *			
HRQOL-Total			−0.49 **			−0.45 **

* *p* < 0.05; ** *p* < 0.01; *** *p* < 0.001.

**Table 7:** Prediction of physical QOL by handwriting measures among adolescents with dysgraphia and the control group.

	Dysgraphia			Control		
Variable	Model 1			Model 1		
	B	SE B	β	B	SE B	β
Stroke height (mm)	−1.490	0.67	−0.32 *	−0.986	0.51	−0.113
Stroke duration (s)	2.630	1.08	0.35 *	1.164	1.45	0.103
R^2^	0.249			0.019		
ΔR^2^	0.209			−0.034		
F change in R^2^	6.140 **			0.352		

* *p* < 0.05; ** *p* < 0.01.

**Table 8:** Prediction of physical QOL by the ADC subscales among adolescents with dysgraphia and the control group.

	Dysgraphia			Control		
	Model 1			Model 1		
	B	SE B	β	B	SE B	β
ADC-C	−1.080	0.277	−0.534 ***	−1.110	0.230	−0.617 ***
R^2^	0.285			0.381		
ΔR^2^	0.266			0.365		
F change in R^2^	15.161 ***			23.410 ***		
	Model 2					
ADC-C	−1.627	0.280	−0.805 ***			
ADC-A	0.715	0.190	0.521 ***			
R^2^	0.484					
ΔR^2^	0.456					
F change in R^2^	14.217 ***					

*** *p* < 0.001.

**Table 9:** Prediction of physical QOL by handwriting measures and ADC subscale among adolescents with dysgraphia.

Variable	Model 1			Model 2			Model 3		
	*B*	SE B	β	B	SE	β	B	SE	β
Stroke height (mm)	−1.67	0.703	−0.36 *	−1.490	0.666	−0.322 *	−0.987	0.641	−0.212
Stroke duration (s)				2.635	1.085	0.348 *	1.812	1.043	−1.540
ADC-C							−0.801	0.291	−0.396
R^2^	0.130			0.249			0.380		
ΔR^2^	0.107			0.209			0.328		
F change in R^2^	5.654 *			6.140 **			7.345 ***		

* *p* < 0.05, ** *p* < 0.01, *** *p* < 0.001.

## Data Availability

The data presented in this study are available on request from the corresponding author. The data are not publicly available due to privacy and ethical reasons.
